# Supplementary material for: Targeted surveillance strategies for efficient detection of novel antibiotic resistance variants
Source: eLife. 2020 Jun 30;9:e56367. doi: 10.7554/eLife.56367 (PMC7326491; doi:10.7554/eLife.56367)
Supplement: Supplementary file 2. — (A) Demographic and geographic sampling biases in datasets 1–3. (B) Detection efficiency of random, demography-, niche-, and geography-aware sampling approaches for resistance variants. (C) Detection efficiency of random sampling, as well as preferential sampling of patients that had recently engaged in overseas sex or in sex work, for resistance variants in dataset 2. (D) Detection efficiency of random and phylogeny-aware sampling approaches for resistance variants. (E) Detection efficiency of random and phylogeny-aware sampling approaches for variants associated with diagnostic escape. (F) Detection efficiency of random and genomic background-aware sampling approaches for resistance variants. [file elife-56367-supp2.docx]

**Supplementary file 2A.** Demographic and geographic sampling biases in datasets 1-3.

|  |  | **Dataset 1 [1]**^a^ | **US**  **(2011-2015) [2-6]** | **Dataset 2 [7]**^a^ | **Victoria, Australia (2017)** | **Dataset 3 [8]** | **Euro-GASP countries (2013) [9]** |
| --- | --- | --- | --- | --- | --- | --- | --- |
| **Proportion of total gonorrhea cases that were from** | men | 0.958 | 0.603 | 0.872 | 0.81 | N/A | N/A |
|  | MSM | 0.737 | 0.295 | 0.733 | 0.567 | N/A | N/A |
|  | Austria | N/A | N/A | N/A | N/A | 0.051 | 0.023 |
|  | Belgium | N/A | N/A | N/A | N/A | 0.052 | 0.021 |
|  | Cyprus | N/A | N/A | N/A | N/A | 0.008 | 0.000 |
|  | Denmark | N/A | N/A | N/A | N/A | 0.052 | 0.017 |
|  | France | N/A | N/A | N/A | N/A | 0.054 | 0.028 |
|  | Germany | N/A | N/A | N/A | N/A | 0.045 | N/A |
|  | Greece | N/A | N/A | N/A | N/A | 0.046 | 0.004 |
|  | Hungary | N/A | N/A | N/A | N/A | 0.046 | 0.031 |
|  | Iceland | N/A | N/A | N/A | N/A | 0.005 | 0.000 |
|  | Italy | N/A | N/A | N/A | N/A | 0.025 | 0.026 |
|  | Latvia | N/A | N/A | N/A | N/A | 0.036 | 0.011 |
|  | Malta | N/A | N/A | N/A | N/A | 0.019 | 0.001 |
|  | Netherlands | N/A | N/A | N/A | N/A | 0.063 | 0.085 |
|  | Norway | N/A | N/A | N/A | N/A | 0.052 | 0.010 |
|  | Portugal | N/A | N/A | N/A | N/A | 0.102 | 0.002 |
|  | Slovakia | N/A | N/A | N/A | N/A | 0.036 | 0.008 |
|  | Slovenia | N/A | N/A | N/A | N/A | 0.051 | 0.001 |
|  | Spain | N/A | N/A | N/A | N/A | 0.110 | 0.068 |
|  | Sweden | N/A | N/A | N/A | N/A | 0.047 | 0.002 |
|  | UK | N/A | N/A | N/A | N/A | 0.101 | 0.661 |

MSM, men who have sex with men

^a^Proportion of patients with identified gender or sexual behavior that identified as men or MSM

**Supplementary file 2B.** Detection efficiency of random, demography-, niche-, and geography-aware sampling approaches for resistance variants.

| **Dataset** | **Variant** | **Sampling approach^a^** | | | | | |
| --- | --- | --- | --- | --- | --- | --- | --- |
|  |  | **Random^b^** | **Demography-aware (M vs. W)** | **Demography-aware (MSM vs. WSM/MSW)** | **Niche-aware** | **Geography- and distance-aware** | **Geography-aware** |
| 1 | RplD G70D | N/A | N/A | N/A | N/A | N/A | N/A |
|  | 23S C2611T (2-4 alleles) | 0.51 (0.46-0.57) | 0.52 (0.46-0.57), *P* = 0.9335, -0.002 | 0.45 (0.4-0.5), *P* = 0.0892,  -0.13 | 0.41 (0.36-0.47), *P* = 0.0103,  -0.1526 | N/A | N/A |
|  | *penA* XXXIV | 0.98 (0.97-0.98) | 0.98 (0.98-0.98), *P* = 0.7405, 0.001 | 0.98 (0.98-0.99), *P* = 0.2383, 0.003 | 0.97 (0.97-0.98), *P* = 0.2446,  -0.006 | N/A | N/A |
| 2 | RplD G70D | 0.96 (0.96-0.97) | 0.96 (0.95-0.97), *P* = 0.4618, -0.007 | 0.95 (0.94-0.96), *P* = 0.0.0202,  -0.02 | 0.96 (0.95-0.97), *P* = 0.2933,  -0.006 | N/A | N/A |
|  | 23S C2611T (2-4 alleles) | 0.97 (0.96-0.97) | 0.97 (0.96-0.97), *P* = 0.3035, -0.004 | 0.96 (0.95-0.97), *P* = 0.5820, 0.001 | 0.96 (0.95-0.97), *P* = 0.4383,  -0.005 | N/A | N/A |
|  | *penA* XXXIV | 0.98 (0.97-0.98) | 0.96 (0.96-0.97), *P* = 0.0013, -0.009 | 0.96 (0.96-0.97), *P* = 0.0013, -0.01 | 0.97 (0.97-0.98), *P* = 0.1836,  -0.004 | N/A | N/A |
| 3 | RplD G70D | 0.96 (0.95-0.97) | N/A | N/A | N/A | 0.96 (0.96-0.97), *P* = 0.2535, 0.004 | 0.96 (0.95-0.96), *P* = 0.3835,  -0.008 |
|  | 23S C2611T (2-4 alleles) | 0.93 (0.91-0.94) | N/A | N/A | N/A | 0.88 (0.86-0.9), *P* = 0.0017,  -0.03 | 0.88 (0.86-0.9), *P* = 0.0018,  -0.04 |
|  | *penA* XXXIV | N/A | N/A | N/A | N/A | N/A | N/A |
| 4 | RplD G70D | N/A | N/A | N/A | N/A | N/A | N/A |
|  | 23S C2611T (2-4 alleles) | 0.75 (0.71-0.79) | N/A | N/A | N/A | 0.67 (0.64-0.71), *P* < 0.0001,  -0.17 | 0.63 (0.6-0.66), *P* = 0.0007,  -0.12 |
|  | *penA* XXXIV | 0.52 (0.46-0.58) | N/A | N/A | N/A | 0.42 (0.37-0.47), *P* = 0.0453,  -0.11 | 0.43 (0.39-0.48), *P* = 0.0172,  -0.14 |

^a^Mean detection efficiency with 95% confidence intervals, *P*-value (by Mann Whitney U test of difference in mean ranks of detection efficiencies between random sampling and the targeted sampling approach based on 100 simulations of each sampling approach), and the difference between median detection efficiencies between the targeted sampling approach and random sampling.

**Supplementary file 2B (cont.).**

^b^Mean detection efficiency with 95% confidence intervals achieved by random sampling from all isolates for which the presence of absence of the variant could be determined. Note, however, that for targeted sampling approaches based on each different patient characteristic, isolates with missing information for that patient characteristic were removed, and random sampling was simulated on the reduced dataset for comparison to the targeted sampling approach.

**Supplementary file 2C.** Detection efficiency of random sampling, as well as preferential sampling of patients that had recently engaged in overseas sex or in sex work, for resistance variants in dataset 2.

| **Variant** | **Sampling approach^a^** | | | |
| --- | --- | --- | --- | --- |
|  | **Random^b^** | **Only patients with recent overseas sex** | **Only sex workers** |  |
| 23S C2611T (2-4 alleles) | 0.96 (0.95-0.97) | 0^c^ | N/A |  |
| *penA* XXXIV | 0.96 (0.96-0.97) | 0.98 (0.98-0.98), *P* = 0.0008, 0.007 | N/A |  |
| RplD G70D | 0.96 (0.95-0.96) | 0.99 (0.98-0.99), *P* < 0.0001, 0.02 | N/A |  |
| 23S C2611T (2-4 alleles) | 0.96 (0.95-0.96) | N/A | 0.98 (0.98-0.98), *P* < 0.0001, 0.01 |  |
| *penA* XXXIV | 0.97 (0.96-0.97) | N/A | 0.98 (0.97-0.98), *P* = 0.2681, 0.002 |  |
| RplD G70D | 0.96 (0.95-0.97) | N/A | 0.97 (0.97-0.98), *P* = 0.1668, 0.001 |  |

^a^Mean detection efficiency with 95% confidence intervals, *P*-value (by Mann Whitney U test of difference in mean ranks of detection efficiencies between random sampling and the targeted sampling approach based on 100 simulations of each sampling approach), and the difference between median detection efficiencies between the targeted sampling approach and random sampling.

^b^Mean detection efficiency with 95% confidence intervals achieved by random sampling from all isolates for which the presence of absence of the variant could be determined and the relevant patient metadata (*i.e.,* overseas vs. local sex or sex worker status) was available.

^c^No isolates with 23S C2611T mutations were from patients with recent overseas sex.

**Supplementary file 2D.** Detection efficiency of random and phylogeny-aware sampling approaches for resistance variants.

| **Dataset** | **Variant** | **Sampling approach^a^** | | | | |  |
| --- | --- | --- | --- | --- | --- | --- | --- |
|  |  | **Random** | **Phylogeny-aware (distance maximization)** | **Phylogeny-aware (clonal group, 134 SNP threshold)** | **Phylogeny-aware (clonal group, 422 SNP threshold)** | **Phylogeny-aware (fastbaps groups)** | **Phylogeny-aware (MLST)** |
| 1 | RplD G70D | N/A | N/A | N/A | N/A | N/A | N/A |
|  | 23S C2611T (2-4 alleles) | 0.51 (0.46-0.57) | 0.54 (0.54-0.54), *P* = 0.7950, -0.005 | 0.71 (0.68-0.74), *P* < 0.0001, 0.17 | 0.87 (0.86-0.89), *P* < 0.0001, 0.30 | 0.54 (0.5-0.58), *P* = 0.7132, -0.07 | 0.33 (0.29-0.37), *P* < 0.0001, -0.29 |
|  | *penA* XXXIV | 0.98 (0.97-0.98) | 0.9 (0.89-0.9), *P* < 0.0001,  -0.09 | 0.99 (0.98-0.99), *P* = 0.0245, 0.003 | 0.99 (0.98-0.99), *P* = 0.0552, 0.002 | 0.97 (0.97-0.98), *P* = 0.1909, -0.005 | 0.97 (0.97-0.98), *P* = 0.0314,  -0.01 |
| 2 | RplD G70D | 0.96 (0.96-0.97) | 0.99 (0.99-0.99), *P* < 0.0001,  0.02 | 0.99 (0.98-0.99), *P* < 0.0001,  0.01 | 0.99 (0.99-0.99), *P* < 0.0001,  0.01 | 0.99 (0.99-0.99), *P* < 0.0001,  0.02 | 0.99 (0.99-0.99), *P* < 0.0001,  0.02 |
|  | 23S C2611T (2-4 alleles) | 0.97 (0.96-0.97) | 0.93 (0.93-0.93), *P* < 0.0001,  -0.04 | 0.98 (0.98-0.99), *P* < 0.0001,  0.01 | 0.99 (0.98-0.99), *P* < 0.0001,  0.01 | 0.97 (0.96-0.97), *P* = 0.6824,  0.003 | 0.97 (0.96-0.97) |
|  | *penA* XXXIV | 0.98 (0.97-0.98) | 0.98 (0.98-0.98), *P* = 0.0011,  -0.006 | 0.99 (0.99-0.99), *P* < 0.0001,  0.008 | 0.99 (0.99-0.99), *P* < 0.0001,  0.009 | 0.99 (0.99-0.99), *P* < 0.0001,  0.006 | 0.98 (0.97-0.98), *P* = 0.3017,  -0.004 |
| 3 | RplD G70D | 0.96 (0.95-0.97) | 0.95 (0.95-0.95), *P* < 0.0001,  -0.02 | 0.97 (0.97-0.97), *P* = 0.1348,  0.003 | 0.97 (0.96-0.97), *P* = 0.0784,  0.007 | 0.98 (0.97-0.98), *P* = 0.0018,  0.009 | 0.93 (0.92-0.94), *P* < 0.0001,  -0.03 |
|  | 23S C2611T (2-4 alleles) | 0.93 (0.91-0.94) | 0.71 (0.71-0.71), *P* < 0.0001,  -0.23 | 0.91 (0.9-0.93), *P* = 0.1000,  -0.02 | 0.92 (0.91-0.93), *P* = 0.0679,  -0.03 | 0.98 (0.98-0.99), *P* < 0.0001,  0.04 | 0.76 (0.74-0.77), *P* < 0.0001,  -0.20 |
|  | *penA* XXXIV | N/A | N/A | N/A | N/A | N/A | N/A |
| 4 | RplD G70D | N/A | N/A | N/A | N/A | N/A | N/A |
|  | 23S C2611T (2-4 alleles) | 0.75 (0.71-0.79) | 0.44 (0.42-0.45), *P* < 0.0001,  -0.35 | 0.64 (0.6-0.69), *P* = 0.0003,  -0.11 | 0.68 (0.64-0.72), *P* = 0.0155,  -0.08 | 0.67 (0.63-0.71) *P* = 0.003,  -0.09 | 0.69 (0.66-0.73), *P* < 0.0001,  -0.08 |
|  | *penA* XXXIV | 0.52 (0.46-0.58) | 0.43 (0.42-0.44), *P* = 0.0497,  -0.11 | 0.48 (0.42-0.53), *P* = 0.4304,  -0.17 | 0.53 (0.48-0.58), *P* = 0.7141,  -0.07 | 0.49 (0.45-0.53), *P* = 0.5604,  -0.10 | 0.44 (0.39-0.49), *P* = 0.1170,  -0.19 |
| 5 | RplD G70D | 0.76 (0.73-0.8) | 0.98 (0.98-0.98), *P* < 0.0001,  0.16 | 0.9 (0.89-0.91), *P* < 0.0001,  0.08 | 0.9 (0.89-0.91), *P* < 0.0001,  0.08 | 0.96 (0.96-0.97), *P* < 0.0001,  0.14 | 0.95 (0.95-0.96), *P* < 0.0001,  0.13 |
|  | 23S C2611T (2-4 alleles) | 0.65 (0.6-0.7) | 0.93 (0.93-0.93), *P* < 0.0001,  0.23 | 0.9 (0.89-0.91), *P* < 0.0001,  0.19 | 0.91 (0.9-0.92), *P* < 0.0001,  0.20 | 0.98 (0.97-0.98), *P* < 0.0001,  0.28 | 0.96 (0.95-0.96), *P* < 0.0001,  0.25 |
|  | *penA* XXXIV | 0.89 (0.87-0.91) | 0.95 (0.95-0.95), *P* < 0.0001,  0.03 | 0.95 (0.94-0.95), *P* < 0.0001,  0.03 | 0.9 (0.89-0.91), *P* < 0.0001,  0.03 | 0.96 (0.96-0.97), *P* < 0.0001,  0.04 | 0.86 (0.84-0.88), *P* = 0.0088,  -0.06 |

**Supplementary file 2D (cont.).**

^a^Mean detection efficiency with 95% confidence intervals, *P*-value (by Mann Whitney U test of difference in mean ranks of detection efficiencies between random sampling and the targeted sampling approach based on 100 simulations of each sampling approach), and the difference between median detection efficiencies between the targeted sampling approach and random sampling.

**Supplementary file 2E.** Detection efficiency of random and phylogeny-aware sampling approaches for variants associated with diagnostic escape.

| **Dataset** | **Variant** | **Sampling approach^a^** | | | | |  |
| --- | --- | --- | --- | --- | --- | --- | --- |
|  |  | **Random** | **Phylogeny-aware (distance maximization)** | **Phylogeny-aware (clonal group, 134 SNP threshold)** | **Phylogeny-aware (clonal group, 422 SNP threshold)** | **Phylogeny-aware (fastbaps groups)** | **Phylogeny-aware (MLST)** |
| 1 | *N. meningitidis*-like porA | 0.52 (0.46-0.58) | 0.52 (0.52-0.52), *P* = 0.1187, -0.04 | 0.86 (0.85-0.87), *P* < 0.0001, 0.29 | 0.7 (0.68-0.73), *P* < 0.0001, 0.12 | 0.74 (0.72-0.77), *P* < 0.0001, 0.16 | 0.31 (0.26-0.35), *P* < 0.0001, -0.35 |
|  | *cppB* deletion | 0.91 (0.9-0.93) | 0.99 (0.99-0.99), *P* < 0.0001, 0.06 | 0.96 (0.95-0.96), *P* < 0.0001, 0.04 | 0.97 (0.96-0.97), *P* < 0.0001, 0.04 | 0.98 (0.98-0.99), *P* < 0.0001, 0.06 | 0.98 (0.98-0.98), *P* < 0.0001, 0.06 |
|  | 16S rRNA | 0.51 (0.45-0.56) | 0.42 (0.42-0.42), *P* = 0.0675, -0.11 | 0.74 (0.71-0.77), *P* < 0.0001, 0.24 | 0.71 (0.68-0.73), *P* < 0.0001, 0.15 | 0.73 (0.71-0.76), *P* < 0.0001, 0.19 | 0.33 (0.29-0.38), *P* < 0.0001, -0.28 |
|  | DR-9A G168A | N/A | N/A | N/A | N/A | N/A | N/A |
| 2 | *N. meningitidis*-like porA | 0.53 (0.48-0.59) | 0.87 (0.87-0.87), *P* < 0.0001, 0.32 | 0.93 (0.93-0.94), *P* < 0.0001, 0.38 | 0.96 (0.95-0.96), *P* < 0.0001, 0.41 | 0.99 (0.99-0.99), *P* < 0.0001, 0.45 | 0.44 (0.40-0.48), *P* = 0.0097, -0.10 |
|  | *cppB* deletion | 0.51 (0.45-0.56) | 1 (1-1), *P* < 0.0001, 0.46 | 0.93 (0.93-0.94), *P* < 0.0001, 0.39 | 0.96 (0.95-0.96), *P* < 0.0001, 0.42 | 0.99 (0.99-0.99), *P* < 0.0001, 0.45 | 0.98 (0.98-0.99), *P* < 0.0001, 0.44 |
|  | 16S rRNA | 0.69 (0.64-0.73) | 0.94 (0.94-0.94), *P* < 0.0001, 0.20 | 0.85 (0.84-0.87), *P* < 0.0001, 0.10 | 0.9 (0.89-0.91), *P* < 0.0001, 0.16 | 0.93 (0.93-0.94), *P* < 0.0001, 0.20 | 0.93 (0.92-0.94), *P* < 0.0001, 0.19 |
|  | DR-9A G168A | 0.69 (0.64-0.73) | 0.93 (0.93-0.93), *P* < 0.0001, 0.20 | 0.95 (0.95-0.96), *P* < 0.0001, 0.22 | 0.97 (0.97-0.97), *P* < 0.0001, 0.24 | 0.68 (0.64-0.71), *P* = 0.4449, -0.05 | 0.98 (0.98-0.98), *P* < 0.0001, 0.25 |
| 3 | *N. meningitidis*-like porA | N/A | N/A | N/A | N/A | N/A | N/A |
|  | *cppB* deletion | 0.85 (0.82-0.87) | 0.99 (0.99-0.99), *P* < 0.0001, 0.11 | 0.91 (0.9-0.92), *P* = 0.0025, 0.03 | 0.94 (0.93-0.94), *P* < 0.0001, 0.06 | 0.99 (0.98-0.99), *P* < 0.0001, 0.10 | 0.97 (0.96-0.97), *P* < 0.0001, 0.09 |
|  | 16S rRNA | N/A | N/A | N/A | N/A | N/A | N/A |
|  | DR-9A G168A | N/A | N/A | N/A | N/A | N/A | N/A |
| 5 | *N. meningitidis*-like porA | N/A | N/A | N/A | N/A | N/A | N/A |
|  | *cppB* deletion | 0.96 (0.96-0.97) | 0.97 (0.97-0.97), *P* = 0.9799, -0.003 | 0.98 (0.97-0.98), *P =* 0.0094, 0.008 | 0.98 (0.97-0.98), *P* = 0.0219, 0.005 | 0.96 (0.95-0.96), *P* = 0.0070,  ‑0.006 | 0.98 (0.97-0.98), *P* = 0.0005, 0.01 |
|  | 16S rRNA | N/A | N/A | N/A | N/A | N/A | N/A |
|  | DR-9A G168A | N/A | N/A | N/A | N/A | N/A | N/A |

**Supplementary file 2E (cont.).**

^a^Mean detection efficiency with 95% confidence intervals, *P*-value (by Mann Whitney U test of difference in mean ranks of detection efficiencies between random sampling and the targeted sampling approach based on 100 simulations of each sampling approach), and the difference between median detection efficiencies between the targeted sampling approach and random sampling.

**Supplementary file 2F.** Detection efficiency of random and genomic background-aware sampling approaches for resistance variants.

| **Dataset** | **Variant** | **Sampling approach^a^** | | |
| --- | --- | --- | --- | --- |
|  |  | **Random** | **Genomic background-aware (only GyrA S91F isolates)** | **Genomic background-aware (only PorB G120/A121 mutation isolates)** |
| 1 | CRO-RS (≥0.12 μg/mL) | 0.91 (0.9-0.93), | 0.98 (0.97-0.98), *P* < 0.0001, 0.05 | 0.96 (0.95-0.97), *P* = 0.0002, 0.03 |
|  | CFX-R (>0.25 μg/mL) | 0.51 (0.45-0.57) | 0.87 (0.85-0.88), *P* < 0.0001, 0.37 | 0.77 (0.74-0.8), *P* < 0.0001, 0.28 |
| 3 | CRO-RS (≥0.12 μg/mL) | 0.91 (0.89-0.93) | 0.95 (0.94-0.96), *P* = 0.0004, 0.02 | 0.94 (0.93-0.95), *P* = 0.0146, 0.02 |
|  | CFX-R (>0.25 μg/mL) | 0.88 (0.86-0.9) | 0.93 (0.92-0.94) *P* = 0.0006, 0.04 | 0.92 (0.9-0.93), *P* < 0.0001, 0.02 |
| 4 | CRO-RS (≥0.12 μg/mL) | 0.93 (0.92-0.94) | 0.95 (0.94-0.96) *P* = 0.0060, 0.02 | 0.96 (0.95-0.97), *P* = 0.0001, 0.03 |
|  | CFX-R (>0.25 μg/mL) | 0.96 (0.95-0.96) | 0.97 (0.96-0.97), *P* = 0.0462, 0.004 | 0.97 (0.97-0.98), *P* = 0.0171, 0.008 |
| 5 | CRO-RS (≥0.12 μg/mL) | 0.5 (0.45-0.56) | 0.85 (0.83-0.87), *P* < 0.0001, 0.36 | 0.83 (0.81-0.85) *P* < 0.0001, 0.31 |
|  | CFX-R (>0.25 μg/mL) | N/A | N/A | N/A |

^a^Mean detection efficiency with 95% confidence intervals, *P*-value (by Mann Whitney U test of difference in mean ranks of detection efficiencies between random sampling and the targeted sampling approach based on 100 simulations of each sampling approach), and the difference between median detection efficiencies between the targeted sampling approach and random sampling.

**Supplementary References:**

1. Mortimer TD, Pathela P, Crawley A, Rakeman JL, Lin Y, Harris SR, et al. The distribution and spread of susceptible and resistant Neisseria gonorrhoeae across demographic groups in a major metropolitan center. medRxiv. 2020. doi: 10.1101/2020.04.30.20086413.

2. Centers for Disease Control and Prevention. Sexually Transmitted Disease Surveillance 2011. Atlanta: U.S. Department of Health and Human Services, 2012.

3. Centers for Disease Control and Prevention. Sexually Transmitted Disease Surveillance 2012. Atlanta: U.S. Department of Health and Human Services, 2013.

4. Centers for Disease Control and Prevention. Sexually Transmitted Disease Surveillance 2013. Atlanta: U.S. Department of Health and Human Services, 2014.

5. Centers for Disease Control and Prevention. Sexually Transmitted Disease Surveillance 2014. Atlanta: U.S. Department of Health and Human Services, 2015.

6. Centers for Disease Control and Prevention. Sexually Transmitted Disease Surveillance 2015. Atlanta: U.S. Department of Health and Human Services, 2016.

7. Williamson D, Chow EPF, Gorrie C, Seemann T, Ingle DJ, Higgins N, et al. Bridging of Neisseria Gonorrhoeae Across Diverse Sexual Networks in the HIV Pre-Exposure Prophylaxis (PrEP) Era: A Clinical and Molecular Epidemiological Study. Nature Communications. 2019;10(1):3988.

8. Harris SR, Cole MJ, Spiteri G, Sanchez-Buso L, Golparian D, Jacobsson S, et al. Public health surveillance of multidrug-resistant clones of Neisseria gonorrhoeae in Europe: a genomic survey. Lancet Infect Dis. 2018;18(7):758-68. doi: 10.1016/S1473-3099(18)30225-1. PubMed PMID: 29776807; PubMed Central PMCID: PMCPMC6010626.

9. European Centre for Disease Prevention and Control. Gonococcal antimicrobial susceptibility surveillance in Europe 2013. Stokholm: ECDC, 2015.
